# 2162. *In Vitro* Antimicrobial Activity of Ceftobiprole against *Streptococcus pneumoniae* Isolates from the United States (2016–2020)

**DOI:** 10.1093/ofid/ofad500.1785

**Published:** 2023-11-27

**Authors:** Leonard R Duncan, Mariana Castanheira, Jennifer Smart, Mark E Jones, Rodrigo E Mendes

**Affiliations:** JMI Laboratories, North Liberty, Iowa; JMI Laboratories, North Liberty, Iowa; Basilea Pharmaceutica International Ltd, Allschwil, Basel-Landschaft, Switzerland; Basilea Pharmaceutica International Ltd., Allschwil, Switzerland, Allschwil,, Basel-Landschaft, Switzerland; JMI Laboratories, North Liberty, Iowa

## Abstract

**Background:**

Ceftobiprole (BPR) is an advanced-generation cephalosporin approved in Europe and many non-European countries for the treatment of community-acquired pneumonia and non–ventilator-associated hospital-acquired pneumonia in adults caused by indicated species, including *Streptococcus pneumoniae* (SPN). This study evaluated the *in vitro* antimicrobial activity of BPR and comparator agents against recent SPN isolates causing lower respiratory tract infections in patients at medical centers in the United States (US).

**Methods:**

A total of 2,793 SPN isolates from 32 US medical centers (2016–2020) were collected from patients with lower respiratory tract infections. Isolates were tested for antimicrobial susceptibility using the Clinical and Laboratory Standards Institute (CLSI) broth microdilution method. MIC interpretations for BPR and comparators utilized European Committee on Antimicrobial Susceptibility Testing (EUCAST) or CLSI criteria, respectively. The EUCAST BPR susceptibility breakpoint for SPN was 0.5 mg/L.

**Results:**

BPR inhibited 99.5% of all SPN isolates at the EUCAST breakpoint (MIC_50/90_, 0.015/0.25 mg/L). The MIC_50/90_ values were stable over all 5 surveillance years. The following comparator agents displayed lower activity against the isolate set: clindamycin (85.5% susceptible [S]), erythromycin (53.2% S), penicillin (63.2% S using oral breakpoints), tetracycline (79.4% S), and trimethoprim-sulfamethoxazole (72.8% S). BPR maintained excellent *in vitro* activity against each of the isolate subsets resistant to these comparator antimicrobials (95.4–98.8% S to BPR depending on the comparator antimicrobial; 95.4% of the penicillin-resistant isolates were S to BPR). This SPN isolate set was also 99.4% S to levofloxacin, 97.4% S to ceftriaxone (using non-meningitis breakpoints), and 100% S to linezolid and vancomycin.

**Conclusion:**

BPR exhibited potent *in vitro* antimicrobial activity against recent SPN clinical isolates collected in US medical centers (2016–2020). These data indicate that BPR merits further investigation as a potential option for the treatment of lower respiratory tract infections caused by SPN in the US.

**Disclosures:**

**Leonard R. Duncan, PhD**, AbbVie: Grant/Research Support|Basilea: Grant/Research Support|CorMedix: Grant/Research Support|Melinta: Grant/Research Support|Pfizer: Grant/Research Support **Mariana Castanheira, PhD**, AbbVie: Grant/Research Support|Basilea: Grant/Research Support|bioMerieux: Grant/Research Support|Cipla: Grant/Research Support|CorMedix: Grant/Research Support|Entasis: Grant/Research Support|Melinta: Grant/Research Support|Paratek: Grant/Research Support|Pfizer: Grant/Research Support|Shionogi: Grant/Research Support **Jennifer Smart, PhD**, Basilea Pharmaceutica International Ltd, Allschwil, Switzerland: Stocks/Bonds **Mark E. Jones, PhD**, Astellas Pharma Global Development, Inc: Support for the present publication|Basilea Pharmaceutica International Ltd: Employee of Basilea Pharmaceutica International Ltd|Basilea Pharmaceutica International Ltd: Stocks/Bonds **Rodrigo E. Mendes, PhD**, AbbVie: Grant/Research Support|Basilea: Grant/Research Support|Cipla: Grant/Research Support|Entasis: Grant/Research Support|GSK: Grant/Research Support|Paratek: Grant/Research Support|Pfizer: Grant/Research Support|Shionogi: Grant/Research Support

